# Application of Two-Dimensional Entropy Measures to Detect the Radiographic Signs of Tooth Resorption and Hypercementosis in an Equine Model

**DOI:** 10.3390/biomedicines10112914

**Published:** 2022-11-13

**Authors:** Kamil Górski, Marta Borowska, Elżbieta Stefanik, Izabela Polkowska, Bernard Turek, Andrzej Bereznowski, Małgorzata Domino

**Affiliations:** 1 Department of Large Animal Diseases and Clinic, Institute of Veterinary Medicine, Warsaw University of Life Sciences, 02-787 Warsaw, Poland; elzbieta_stefanik@sggw.edu.pl (E.S.); bernard_turek@sggw.edu.pl (B.T.); 2 Institute of Biomedical Engineering, Faculty of Mechanical Engineering, Białystok University of Technology, 15-351 Bialystok, Poland; m.borowska@pb.edu.pl; 3 Department and Clinic of Animal Surgery, Faculty of Veterinary Medicine, University of Life Sciences, 20-950 Lublin, Poland; izabela.polkowska@up.lublin.pl; 4 Division of Veterinary Epidemiology and Economics, Institute of Veterinary Medicine, Warsaw University of Life Sciences, Nowoursynowska 159c, 02-776 Warsaw, Poland; andrzej_bereznowski@sggw.edu.pl

**Keywords:** radiographs, texture analysis, entropy–based approaches, equine odontoclastic tooth resorption and hypercementosis, dental care

## Abstract

Dental disorders are a serious health problem in equine medicine, their early recognition benefits the long-term general health of the horse. Most of the initial signs of Equine Odontoclastic Tooth Resorption and Hypercementosis (EOTRH) syndrome concern the alveolar aspect of the teeth, thus, the need for early recognition radiographic imaging. This study is aimed to evaluate the applicability of entropy measures to quantify the radiological signs of tooth resorption and hypercementosis as well as to enhance radiographic image quality in order to facilitate the identification of the signs of EOTRH syndrome. A detailed examination of the oral cavity was performed in eighty horses. Each evaluated incisor tooth was assigned to one of four grade–related EOTRH groups (0–3). Radiographs of the incisor teeth were taken and digitally processed. For each radiograph, two–dimensional sample (SampEn2D), fuzzy (FuzzEn2D), permutation (PermEn2D), dispersion (DispEn2D), and distribution (DistEn2D) entropies were measured after image filtering was performed using Normalize, Median, and LaplacianSharpening filters. Moreover, the similarities between entropy measures and selected Gray–Level Co–occurrence Matrix (GLCM) texture features were investigated. Among the 15 returned measures, DistEn2D was EOTRH grade–related. Moreover, DistEn2D extracted after Normalize filtering was the most informative. The EOTRH grade–related similarity between DistEn2D and Difference Entropy (GLCM) confirms the higher irregularity and complexity of incisor teeth radiographs in advanced EOTRH syndrome, demonstrating the greatest sensitivity (0.50) and specificity (0.95) of EOTRH 3 group detection. An application of DistEn2D to Normalize filtered incisor teeth radiographs enables the identification of the radiological signs of advanced EOTRH with higher accuracy than the previously used entropy–related GLCM texture features.

## 1. Introduction

Dental disorders, including complications related to cases of oral cavity disease, constitute a serious health problem in equine medicine [1]. As equine hypsodont teeth slowly erupt over most of the horse’s life [2], simple dental rasping is able to improve the welfare and possibly food digestion as well as biting behaviour of more than 70% of horses presenting dental disorders [3]. Thus, dental disorders are of major importance in equine veterinary practice, with up to 10% of practice time involving dental–related work [1].

The current standard of care in equine dentistry includes performing a complete visual oral examination, including general and dental history taking, observation and general physical examination, as well as comprehensive oral and dental examination [4]. However, radiography and possibly other imaging of dental apices and reserve crowns is essential in the evaluation of primary incisor and canine teeth disease affected by resorption and hypercementosis of the reserve crown [5,6], secondary periodontal disease with remodelling and lysis of the alveolar bone [1,7,8], traumatic disorders of teeth [1,9,10], as well as apical infections such as cheek teeth apical abscessation [1,9]. The welfare of the horse depends on skilled and knowledgeable veterinarians who can characterize normal and abnormal findings relative to the oral cavity [4], including frequently occurring dental disorders [1]. Therefore, the digital processing of dental radiographs is proposed here to enhance the quality of the image so that the veterinarians can more easily identify the radiographic signs of disease. As radiography is useful in the diagnosis of tooth resorption and hypercementosis [5,6], bone remodelling and lysis [1,7,8], as well as tooth fractures [1,9,10] and infections [1,9], these preliminary studies focus on the example of Equine Odontoclastic Tooth Resorption and Hypercementosis (EOTRH) syndrome. EOTRH requires radiographic imaging in the early stages to visualize the alveolar aspect of the teeth, where the signs are typically more advanced than is suggestive by the external appearance of the teeth [5,11]. Moreover, the radiographic signs of EOTRH appear earlier than the clinical signs [12,13,14], as 88% of horses with no apparent clinical signs demonstrated radiographic signs of incisor teeth resorption and 20% of horses demonstrated signs of incisor teeth hypercementosis [5]. Resorption and hypercementosis are two ongoing processes that progressively affect the structure of the tooth starting from the alveolar aspect (root and the reserve crown) [15]. Both processes are radiographically well–defined, as teeth demonstrate high radiodensity [16], where low radiopaque signs of resorption and high radiopaque signs of hypercementosis are alternately visible, thus, presenting the high irregularity and complexity of the image texture of radiographs [17].

The complete visual examination of the oral cavity allows the veterinarian to identify and manage dental problems and diagnose early stages of disease, benefiting the long–term general health of the horse and improving its quality of life [4], whereas the main aim of using digital processing of digital radiographs is to enhance the automated detection of early signs of EOTRH disease that might be missed by solely visual evaluation. Thus, the introduction of digital processing to the equine dental radiographs aims to improve the detection of specific signs of dental diseases which are not recognizable during the preliminary visual examination. It has been shown that raw radiographs collected directly from the X–ray scanner, which have not been digitally processed, are less effective in incisor teeth radiographic texture quantification than those that have been digitally processed. Due to the presence of noise, the radiographic filtering by filters improves the edge delimitation, such as Normalize or Bilateral filters, which increases the recognition of the radiographic signs of EOTRH syndrome [17]. However, the specific detection of grade 1, grade 2, and grade 3 EOTRH radiological signs remains challenging.

With the rapid advances in diagnostic imaging in equine dentistry [17,18,19], high–resolution detailed digital images [20] as well as a vast and ever–growing amount of data [17] are more readily available. Digital image processing is increasingly used to select and provide diagnostically important data [21] to avoid information noise that is difficult to evaluate. As the development of the computer–aided detection of image differences is a multi–stage process [22,23], the first two steps towards achieving the main goal have already been attained. The first step, concerning the preliminary demonstration of the possibility of quantifying the texture features of horses’ incisor radiographs, has been previously published [17]. The second step is presented in this study. This second step concerns the advancement in image regularity evaluation, comparison of the novel and recent image regularity indicators, and demonstration of the detection accuracy of radiographic signs of teeth resorption and hypercementosis in both novel and recent experiments. To achieve this intermediate goal, the two–dimensional entropy measures [24], which are relatively recent methods of image irregularity and complexity quantification, have been proposed to obtain relevant and insightful data from equine dental radiographs, which could be applied in early EOTRH diagnosis. As entropy–based measurements represent a new class of easy–to–implement methods [25], their evaluation in different types of clinical applications [26] is advisable. In equine medicine, the entropy–based measurements have been successfully applied for the analysis of the texture of equine thermal images in pregnancy determination [27] and the rider:horse body weight ratio detection in horseback riding [28]. In the study examining the pregnancy model, four entropy–based measurements were used [27], however, in the horses’ back load assessment model, a fifth measurement was added [28]. These five entropy–based measurements included: two–dimensional sample entropy (SampEn2D) [29], two–dimensional fuzzy entropy (FuzzEn2D) [30], two–dimensional permutation entropy (PermEn2D) [31], two–dimensional dispersion entropy (DispEn2D) [32], and two–dimensional distribution entropy (DistEn2D) [33]. These five aforementioned entropy–based measurements have been implemented in the current study. We hypothesize that the quantitative description of incisor tooth radiographs, by using filtrations and entropy measurement extraction, imply the specific detection of the radiological signs of grade 1, grade 2, and grade 3 EOTRH syndrome, which could be used in the future development of the automated detector of early signs of the disease.

Therefore, the study aimed to evaluate the applicability of entropy measures to quantify the radiological signs of tooth resorption and hypercementosis and to enhance the quality of the radiograph to improve the identification of the radiographic signs of EOTRH syndrome. To achieve this goal, the measures of five entropy–based measurements were extracted from radiographs of equine incisor teeth. Then, combinations of measures and filters were used to identify changes associated with the EOTRH grades. Next, the selected entropy measures and filters were compared with recently reported features of gray–level matrices texture analysis approaches to find similarities. Finally, for the selected measures and features, the detection accuracy of the radiographic signs of EOTRH syndrome was calculated.

## 2. Materials and Methods

### 2.1. Horses

Eighty privately owned horses (*n* = 80) (age mean ± SD: 16.9 ± 7.0; 37 geldings, 43 mares; 30 Polish Halfbred horses, 13 Arabian horses, 10 Schlesisches Warmblood horses, 8 Wielkopolska breed horses, 7 Dutch Warmblood horses, 5 Thoroughbred horses, 4 Polish coldblooded horses, and 3 Malopolska breed horses) were enrolled in the current study. The owners presented their horses for the standard veterinary diagnostic procedure including a basic clinical examination [34], a detailed examination of the oral cavity [35], and an additional examination [5]. In the current study, examination data collected from July 2021 to December 2021 were used.

A basic clinical examination was conducted following standard protocol [34], which allowed for the collection of the internal temperature, heart rate, respiratory rate, mucous membranes, capillary refill time, and lymph node evaluation. After a basic clinical examination–based qualification prior to the sedation procedure, the horses received a dose of detomidine hydrochloride (Domosedan; Orion Corporation, Espoo, Finland; 0.01 mg/kg bwt i.v.), xylazine hydrochloride (Xylapan; Vetoquinol Biowet Sp. z o.o., Gorzów Wielkopolski, Poland; 0.4 mg/kg bwt i.v.), or a combination of both. In some cases, the additional dose of butorphanol tartrate (Torbugesic; Zoetis Polska Sp. z o.o., Warsaw, Poland.; 0.01 mg/kg bwt i.v.) was required. No clinical contraindication to the sedation procedure was found in any of the examined horses.

**Figure 1:**
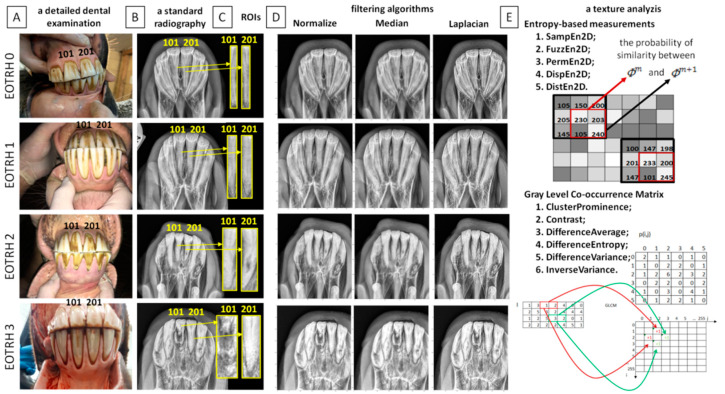
Scheme of radiographic–based detection of the signs of the Equine Odontoclastic Tooth Resorption and Hypercementosis (EOTRH) syndrome. A detailed dental examination (**A**); a standard radiography (**B**); segmentation of input radiographs with regions of interest (ROIs) of the first upper right incisor tooth (101) and the first upper left incisor tooth (201) marked with yellow lines (**C**); filtering of input radiographs by three filters—Normalize, Median, and Laplacian Sharpening (**D**); a texture analysis of output radiographs after filtering using entropy–based measures (five measures: SampEn2D—two–dimensional sample entropy, FuzzEn2D—two–dimensional fuzzy entropy, PermEn2D—two–dimensional permutation entropy, DispEn2D—two–dimensional dispersion entropy, DistEn2D—two–dimensional distribution entropy) and Gray–Level Co–occurrence Matrix (six selected features: Cluster Prominence, Contrast, Difference Average, Difference Entropy, Difference Variance, Inverse Variance) (**E**).

A detailed examination of the oral cavity was conducted following standard protocol and included a visual examination and digital palpation of the teeth [35]. The Haussmann’s mouth speculum was used to ensure the safety of the horse and veterinarian during a detailed examination. A 400 mL syringe was used to flush the oral cavity in order to remove any food which remained on, around, and between teeth. The periodontal probe was used for the evaluation of the interdental spaces. All clinical signs, including the condition of teeth, interdental spaces, gums, and mucosa of the cheeks and tongue, were documented on the equine dental chart [15] (Figure 1A).

An additional examination allowed for radiography of the incisor teeth [5]. The radiographs were obtained by intraoral presentation, which required insertion of the protected radiographic cassette into the opened oral cavity of the horse, following the guidelines of the bisecting angle technique [13]. The dorsoventral projection for the maxillary teeth [15] was achieved by using the following settings: 2.5 mAs; 65 kV; of the x–ray tube (Orange 9020HF, Ecoray Co., Ltd.; 3F, Urbanlight B/D, 630, Eonju–ro, Gangnam–gu, Seoul, Korea) and the same distance (80 cm) to the radiographic cassette (Saturn 8000, Vievorks Co., Ltd., 41–3, Burim–ro, 170beon–gil, Dongan–gu, Anyang–si, Gyeonggi–do, 14055 Korea. The radiographs were acquired on HP portable computer (HP Inc UK Ltd., Earley West, 300 Thames Valley Park Drive, UK) using DxWorks software (Vievorks Co., Ltd., 41–3, Burim–ro, 170beon–gil, Dongan–gu, Anyang–si, Gyeonggi–do, 14055 Korea) and saved as .jpg files (Figure 1B).

The study was approved by the II Local Ethical Committee on Animal Testing in Warsaw on behalf of the National Ethical Committees on Animal Testing (No WAW2/091/2020 approved on 29 July 2020). The owners agreed to use the horses’ data in the current study.

### 2.2. Radiographs Classification

Based on the clinical and radiological signs achieved from the standard veterinary diagnostic procedure, four grade–related EOTRH groups (0–3) were annotated. The radiological classification system introduced by Hüls et al. [36] and modified by Rehl et al. [5] was used. This classification system includes the evaluation of shape, surface structure, contour, and consistency of incisor teeth as well as contour, radiodensity, and delineation of the periodontal space of incisor teeth. As the preliminary application of two–dimensional entropy measures in the radiographic–based detection of the signs of EOTRH syndrome was tested, the central maxillary incisor teeth showing the best presentation in the radiographs obtained in the dorsoventral projection and the lowest superimposition of rounding tissues, were selected for testing. Following the modified Triadan system for equine dental nomenclature [37], the test incisor teeth were numbered as 101 (the first upper right incisor tooth) and 201 (the first upper left incisor tooth). On each selected incisor tooth, the representative rectangular region of interest (ROI) was manually annotated. Each ROI covers the largest possible area of the tooth crown and the largest possible area of the tooth root. Each ROI was individually fitted to the consecutive, separate teeth as shown on Figure 1C. Each ROI was edged by four high–radiodensity lines representing: (i) the occlusal side of the incisor tooth, (ii) the medial side of the incisor tooth, (iii) the apical side of the incisor tooth, and (iv) the lateral side of the incisor tooth, respectively. The ROIs were annotated using the ImageJ software version 1.46r (Wayne Rasband, National Institutes of Mental Health, Bethesda, MD, USA) and saved as .png files.

Based on above criteria, 101 and 201 maxillary incisor teeth were classified to grade 0 (normal teeth; *n* = 37), grade 1 (mildly EOTRH affected teeth; *n* = 94), grade 2 (moderately EOTRH affected teeth; *n* = 20), and grade 3 (severely EOTRH affected teeth; *n* = 8). The total number of incisors for all groups was 159; as 1 incisor was excluded due to tooth fractures.

### 2.3. Digital Radiograph Processing

Radiographs were digitally processed in two steps including input image filtering (Figure 1D) and output image texture analysis (Figure 1E). For the image filtering, three filtering algorithms were chosen: Normalize, Median, and Laplacian Sharpening based on the previously described findings of equine incisor teeth radiographs evaluation [17]. For the image texture analysis, five entropy–based texture measures were considered: two–dimensional sample entropy (SampEn2D), two–dimensional fuzzy entropy (FuzzEn2D), two–dimensional permutation entropy (PermEn2D), two–dimensional dispersion entropy (DispEn2D), and two–dimensional distribution entropy (DistEn2D), based on the previously described applicability in the equine image evaluation [27,28]. Both processing steps were conducted one after the other for the annotated ROIs, so that for each ROI, fifteen filtering–entropy combinations were returned. Each ROI was considered separately. Additionally, the currently presented entropy–based texture measures were compared with the selected, previously reported Gray–Level Co–occurrence Matrix data [17].

One may observe in Górski et al. [17] that the data from the combination of nine filtering algorithms (Mean, Median, Normalize, Bilateral, Binomial, Curvature Flow, Laplacian Sharpening, Discrete Gaussian, and Smoothing Recursive Gaussian) and six texture analysis approaches (First Order Statistics (FOS), Gray–Level Co–occurrence Matrix (GLCM), Neighbouring Gray Tone Difference Matrix (NGTDM), Gray–Level Dependence Matrix (GLDM), Gray–Level Run Length Matrix (GLRLM), and Gray–Level Size Zone Matrix (GLSZM) were returned using PyRadiomics—an open–source python package for the extraction of features from radiographic images [38]—and presented in relation to the radiological signs of EOTRH syndrome grades. The raw data of six selected GLCM features selected in a previous study [17] (Cluster Prominence, Contrast, Difference Average, Difference Entropy, Difference Variance, Inverse Variance) were used in the current study to find similarities with the current raw data of entropy–based texture measures. For the details of the protocol of GLCM features extraction, see Górski et al. [17].

#### 2.3.1. Filtering

Three filtering algorithms were implemented to reduce the noise in the radiographs using SimpleITK toolkit in Python language [39,40]. The used filtering algorithms differ depending on the linearity of the filter, type of output image, and result of filtering, as shown in Table 1.

#### 2.3.2. Extraction of the Entropy–Based Measures

The five entropy–based texture analyses were conducted separately, returning five entropy measures, using Python, version 3.8.5 64–bit using package EntopyHub [43]. The extracted entropy measures differed depending, i.e., on the definition, relations between the values and the irregularity/complexity of the image, and application of measures, as shown in Table 2. For more details and equations of the entropy measures extraction, see Domino et al. [28].

### 2.4. Statistical Analysis

Five entropy measures (SampEn2D, FuzzEn2D, PermEn2D, DispEn2D, DistEn2D) and six selected GLCM features (Cluster Prominence, Contrast, Difference Average, Difference Entropy, Difference Variance, Inverse Variance) were presented as a data series, where each tooth of each horse represented one realization. The entire data set was divided into four EOTRH grade–related groups, thus, four EOTRH grade–labelled data series were extracted. The extracted data series were tested independently for univariate distributions using a Shapiro–Wilk normality test.

EOTRH grade–labelled data series were then compared between EOTRH grades using the ordinary one–way ANOVA followed by Tukey’s multiple comparisons test for Gaussian data and the Kruskal–Wallis test, followed by the Dunn’s multiple comparisons test for non–Gaussian data, and for each entropy measure and GLCM feature independently. The alpha value was established as α = 0.05. On the respective plot, when a measure value was found to significantly increase with the EOTRH grades, the red line was additionally marked. The entropy measures and GLCM features were presented on scatter plots with bars using mean ± SD and dots representing each realization, where lower case letters indicate differences between EOTRH grades.

EOTRH grade–labelled data series were then compared between used filtering algorithms using the ordinary one–way ANOVA followed, by Tukey’s multiple comparisons test for Gaussian data and the Kruskal–Wallis test, followed by the Dunn’s multiple comparisons test for non–Gaussian data, and for each entropy measure and GLCM feature independently. The alpha value was established as α = 0.05. The entropy measures and GLCM features were presented on scatter plots with bars using mean ± SD and dots representing each realization, where lower case letters indicate differences between filtering algorithms.

Based on the received differences, only for the most EOTRH–related entropy measures and GLCM features were linear regressions calculated. On regression plots, two regression equations were displayed, including one selected entropy measure and one for Cluster Prominence, Contrast, Difference Average, Difference Entropy, Difference Variance, or Inverse Variance. Equations were supported with the measure of the difference of linearity. All the slopes were significantly non–zero (*p* < 0.0001). For non–significant differences between the slopes (*p* > 0.05), one slope was calculated and the intercepts were compared. For non–significant differences between the intercepts (*p* > 0.05), one intercept was calculated. When the slope value of the entropy measure was higher than the slope value of the GLCM features, the plot was additionally marked by dashed frames. GraphPad Prism6 software (GraphPad Software Inc., San Diego, CA, USA) was used for all statistical analyses.

Based on the received differences, for the most EOTRH–related entropy measures and GLCM features only, the detection accuracy of EOTRH 0 and EOTRH 3 was calculated using three thresholds for gradually increasing measures (mean, mean + SD, mean + 2SD). The radiograph was annotated as EOTRH 0 when the individually measured value was above the threshold and annotated as EOTRH 3 when below it. The same annotation was carried out for the both EOTRH grade–related groups. The sensitivity (Se), specificity (Sp), positive predictive value (PPV), and negative predictive value (NPV) were estimated. The values of Se, Sp, PPV, and NPV were calculated across the range from 0.1 to 1.0 using standard formulae [48].

## 3. Results

**Figure 2:**
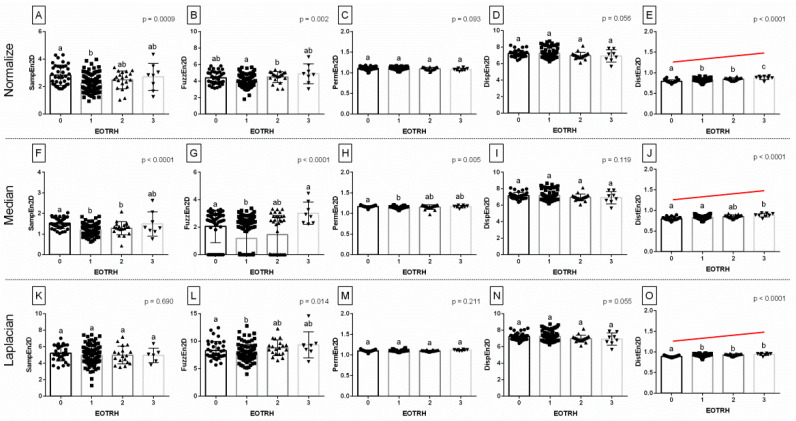
The comparison of the entropy measures between the EOTRH grades (0–3). The following entropy measures are considered: SampEn2D—two–dimensional sample entropy (**A**,**F**,**K**), FuzzEn2D—two–dimensional fuzzy entropy (**B**,**G**,**L**), PermEn2D—two–dimensional permutation entropy (**C**,**H**,**M**), DispEn2D—two–dimensional dispersion entropy (**D**,**I**,**N**), DistEn2D—two–dimensional distribution entropy (**E**,**J**,**O**). The output radiographs filtered by Normalize (**A**–**E**), Median (**F**–**J**), and Laplacian Sharpening (**K**–**O**) filtering algorithms are separated by dashed horizontal lines. Lower case letters (a–c) indicate differences between groups for *p* < 0.05 independently for each measure. The significant increase with the EOTRH grades is marked with a red line. Single realizations are marked with dots.

Among the 15 returned combinations of entropy measures (*n* = 5) and filtering algorithms (*n* = 3), three entropy measures for Normalize filtering output radiographs, four entropy measures for Median filtering output radiographs, and two entropy measures for Laplacian Sharpening filtering output radiographs differed significantly between the EOTRH grades (Figure 2). Although some EOTRH grade–related differences were noted for SampEn2D (Normalize filtering, *p* = 0.0009, Figure 2A and Median filtering, *p* < 0.0001, Figure 2F), FuzzEn2D (Normalize filtering, *p* = 0.002, Figure 2B; Median filtering, *p* < 0.0001, Figure 2G; and Laplacian Sharpening filtering, *p* = 0.0014, Figure 2L), and PermEn2D (Median filtering, *p* = 0.005, Figure 2H), only DistEn2D significantly increased with the EOTRH grades. DistEn2D extracted from the Normalize filtering output radiographs was the lowest in the EOTRH 0 group, higher in the EOTRH 1 and 2 groups, and the highest in the EOTRH 3 group, with no differences between the EOTRH 1 and 2 groups (*p* < 0.0001, Figure 2E). DistEn2D extracted from the Median filtering output radiographs was lower in the EOTRH 0 group compared to the EOTRH 3 group, with no differences between the EOTRH 0–2 groups and EOTRH 2–3 groups (*p* < 0.0001, Figure 2J). DistEn2D extracted from Laplacian Sharpening filtering output radiographs was lower in the EOTRH 0 group compared to EOTRH 1–3 groups with no differences between EOTRH 1–3 groups (*p* < 0.0001, Figure 2O). The significance of this step was to extract entropy–based measurements from the radiographs of equine incisor teeth, and select those combinations of measures and filters that changed with the EOTRH grades.

**Figure 3:**
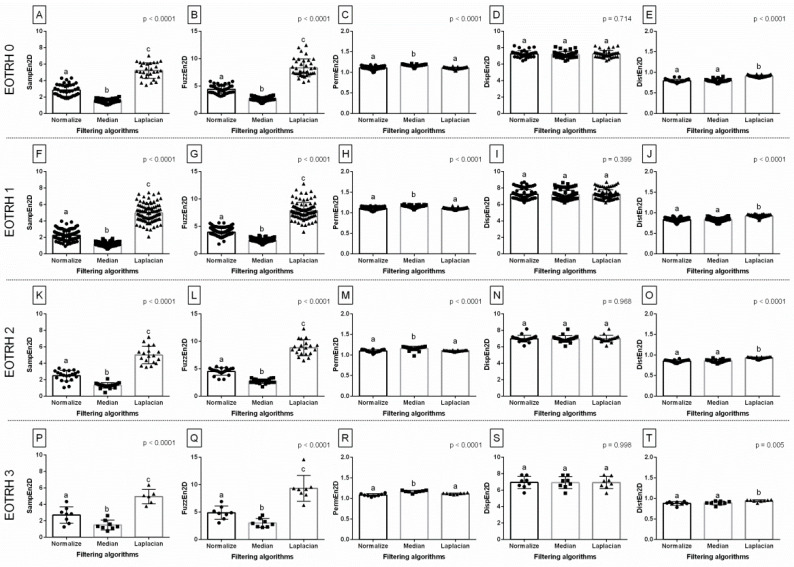
The comparison of the entropy measures between the filtering algorithms. The following entropy measures are considered: SampEn2D—two–dimensional sample entropy (**A**,**F**,**K**,**P**), FuzzEn2D—two–dimensional fuzzy entropy (**B**,**G**,**L**,**Q**), PermEn2D—two–dimensional permutation entropy (**C**,**H**,**M**,**R**), DispEn2D—two–dimensional dispersion entropy (**D**,**I**,**N**,**S**), DistEn2D—two–dimensional distribution entropy (**E**,**J**,**O**,**T**). The radiographs classified to EOTRH 0 grade (**A**–**E**), EOTRH 1 grade (**F**–**J**), EOTRH 2 grade (**K**–**O**), and EOTRH 3 grade (**P**–**T**) are separated by dashed horizontal lines. Lower case letters (a–c) indicate differences between groups for *p* < 0.05 independently for each measure. Single realizations are marked with dots.

When comparing entropy measures between the filtering algorithms, the same differences in consecutive EOTRH groups were noted for SampEn2D (*p* < 0.0001, Figure 3A,F,K,P), FuzzEn2D (*p* < 0.0001, Figure 3B,G,L,Q), PermEn2D (*p* < 0.0001, Figure 3C,H,M,R), and DistEn2D (*p* < 0.0001, Figure 3E,J,O,T), respectively, but not for DispEn2D (*p* > 0.05, Figure 3D,I,N,S). In each EOTRH group, SampEn2D and FuzzEn2D were always the lowest after Median filtering, higher after Normalize filtering, and the highest after Laplacian Sharpening filtering. Similarly, in each EOTRH group, PermEn2D was higher after Median filtering than after Normalize and Laplacian Sharpening filtering, whereas DistEn2D was higher after Laplacian Sharpening filtering than after Normalize and Median filtering. This step demonstrates how the use of filtering algorithms affects the values of the entropy–based measurements received from the consecutive EOTRH groups.

**Figure 4:**
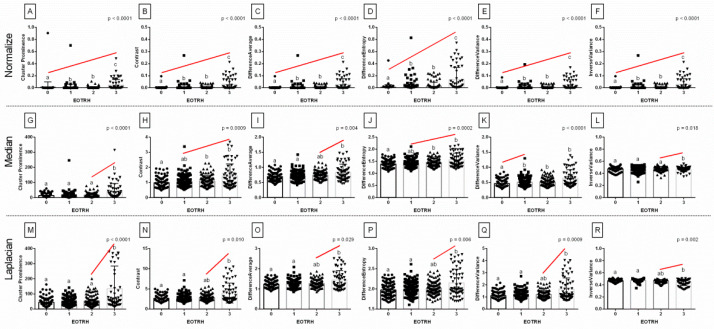
The comparison of the selected Gray–Level Co–occurrence Matrix (GLCM) features between the EOTRH grades (0–3). The following GLCM features are considered: Cluster Prominence (**A**,**G**,**M**), Contrast (**B**,**H**,**N**), Difference Average (**C**,**I**,**O**), Difference Entropy (**D**,**J**,**P**), Difference Variance (**E**,**K**,**Q**), Inverse Variance (**F**,**L**,**R**). The output radiographs filtered by Normalize (**A**–**F**), Median (**G**–**L**), and Laplacian Sharpening (**M**–**R**) filtering algorithms are separated by dashed horizontal lines. Lower case letters (a–c) indicate differences between groups for *p* < 0.05 independently for each feature. The significant increase with the EOTRH grades is marked with a red line. Single realizations are marked with dots.

Among the 18 returned combinations of selected GLCM features (*n* = 6) and filtering algorithms (*n* = 3), all combinations differed significantly between the EOTRH grades and significantly increased with the EOTRH grades (Figure 4). Although some EOTRH grade–related increases were found between EOTRH 0 and 1 groups (Difference Variance after Median filtering, *p* < 0.0001, Figure 4K), between EOTRH 2 and 3 groups (Cluster Prominence after Median filtering, *p* < 0.0001, Figure 4G and Laplacian Sharpening filtering, *p* < 0.0001, Figure 4M; Contrast after Laplacian Sharpening filtering, *p* = 0.010, Figure 4N; Difference Average after Median filtering, *p* = 0.004, Figure 4I and Laplacian Sharpening filtering, *p* = 0.029, Figure 4O; Difference Entropy after Laplacian Sharpening filtering, *p* = 0.006, Figure 4P; Difference Variance after Laplacian Sharpening filtering, *p* = 0.0009, Figure 4Q; Inverse Variance after Median filtering, *p* = 0.018, Figure 4L and Laplacian Sharpening filtering, *p* = 0.002, Figure 4R), and EOTRH 1 and 3 groups (Contrast after Median filtering, *p* = 0.0009, Figure 4H; Difference Entropy after Median filtering, *p* = 0.0002, Figure 4J), all GLCM features significantly increased with the EOTRH grades from EOTRH 0 to 3 after Normalize filtering (*p* < 0.0001, Figure 4A–F). All examined GLCM features extracted from Normalize filtering output radiographs were the lowest in the EOTRH 0 group, higher in the EOTRH 1 and 2 groups, and the highest in the EOTRH 3 group, with no differences between the EOTRH 1 and 2 groups (*p* < 0.0001, Figure 4A–F). This step extracted the GLCM features from the radiographs of equine incisor teeth, and selected those combinations of features and filters that changed with each EOTRH grade.

**Figure 5:**
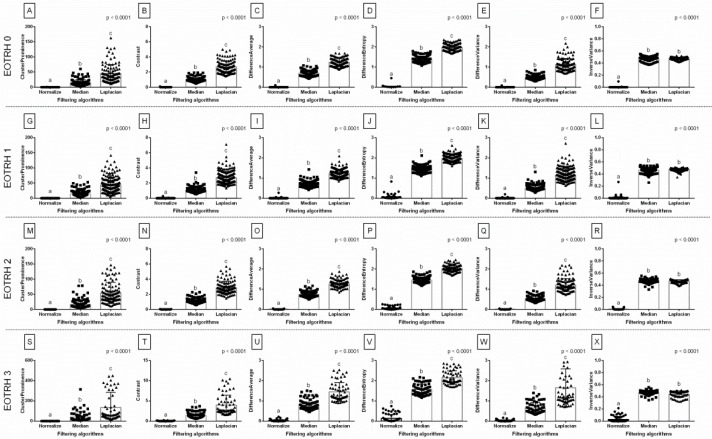
The comparison of the selected Gray–Level Co–occurrence Matrix (GLCM) features between the filtering algorithms. The following GLCM features are considered: Cluster Prominence (**A**,**G**,**M**,**S**), Contrast (**B**,**H**,**N**,**T**), Difference Average (**C**,**I**,**O**,**U**), Difference Entropy (**D**,**J**,**P**,**V**), Difference Variance (**E**,**K**,**Q**,**W**), Inverse Variance (**F**,**L**,**R**,**X**). The radiographs classified to EOTRH 0 grade (**A**–**F**), EOTRH 1 grade (**G**–**L**), EOTRH 2 grade (**M**–**R**), and EOTRH 3 grade (**S**–**X**) are separated by dashed horizontal lines. Lower case letters (a–c) indicate differences between groups for *p* < 0.05 independently for each measure. Single realizations are marked with dots.

When comparing selected GLCM features between the filtering algorithms, the same differences in consecutive EOTRH groups were noted for Cluster Prominence (*p* < 0.0001, Figure 5A,G,M,S), Contrast (*p* < 0.0001, Figure 5B,H,N,T), Difference Average (*p* < 0.0001, Figure 5C,I,O,U), Difference Entropy (*p* < 0.0001, Figure 5D,J,P,V), Difference Variance (*p* < 0.0001, Figure 5E,K,Q,W), and Inverse Variance (*p* < 0.0001, Figure 5F,L,R,X), respectively. In each EOTRH group, Cluster Prominence, Contrast, Difference Average, Difference Entropy, and Difference Variance were always the lowest after Normalize filtering, higher after Median filtering, and highest after Laplacian Sharpening filtering. Similarly, in each EOTRH group, Inverse Variance was higher after Median and Laplacian Sharpening filtering than after Normalize filtering. This demonstrated the effects that the filtering algorithms had on the select GLCM features received from the consecutive EOTRH groups.

**Figure 6:**
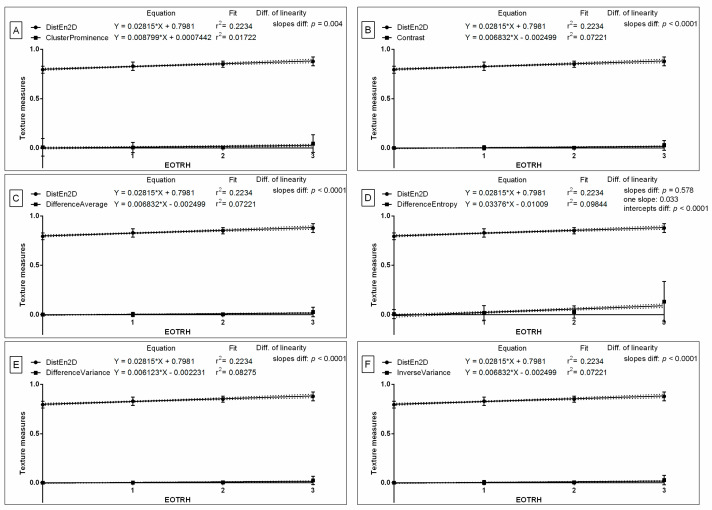
Comparison of selected entropy measure (DistEn2D–two–dimensional distribution entropy) and selected Gray–Level Co–occurrence Matrix (GLCM) features (Cluster Prominence (**A**), Contrast (**B**), Difference Average (**C**), Difference Entropy (**D**), Difference Variance (**E**), Inverse Variance (**F**)) throughout the EOTRH grades. Measure and features were extracted from the output radiographs after Normalize filtering. Similarity was tested using linear regressions. A *p* < 0.05 was considered significant. If the difference between slopes was not significant, a single slope measurement was calculated. Plot where the slope value of the entropy measure was higher than the slope value of the GLCM features was marked by dashed frames.

Based on the received differences, the similarities were tested for DistEn2D and all six GLCM features, of which were only extracted from Normalize filtering output radiographs. The slope of the linear regression equations for DistEn2D compared to the slopes of GLCM features were not significantly different, and one slope measurement was calculated only for Difference Entropy (*p* = 0.578; one slope = 0.033; Figure 6D). The intercept within this data pair was compared and considered significant (*p* < 0.0001), thus, one intercept was not calculated. For all the other compared data pairs, the slopes were significantly different (*p* < 0.05; Figure 6A–C,E,F). The slope value of DistEn2D (slope = 0.028) was higher than the slope value of GLCM features for Cluster Prominence (slope = 0.009; Figure 6A), Contrast (slope = 0.007; Figure 6B), Difference Average (slope = 0.007; Figure 6C), Difference Entropy (slope = 0.006; Figure 6E), and Inverse Variance (slope = 0.007; Figure 6F); but not for Difference Entropy (slope = 0.034; Figure 6D). Here, similarities between the selected entropy measures and GLCM features after subsequent selected types of filtering can be observed.

Based on the received differences, the detection accuracy of EOTRH 0 and EOTRH 3 was tested for DistEn2D and all six GLCM features extracted from Normalize filtering output radiographs (Table 3). For DistEn2D and all six GLCM features, a salient observation was made, identifying that the Se and NPV decreased with higher threshold values (mean > mean + SD > mean + 2SD) and the Sp and PPV increased with higher threshold values (mean > mean + SD > mean + 2SD). For the first threshold (mean), Se ranged from 0.50 for DistEn2D; 0.27 for Difference Entropy; to 0.25 for the remaining five GLCM features. Additionally, Sp ranged from 0.95 for DistEn2D to 0.99 for all GLCM features. For the second threshold (mean + SD), Se ranged from 0.13 for DistEn2D; to 0.22 for Difference Entropy; through 0.17 for the remaining five GLCM features. Moreover, Sp ranged from 1.00 for DistEn2D; to 0.98 for Difference Entropy; through to 0.99 for the remaining five GLCM features. For the third threshold (mean + SD), Se ranged from 0.00 for DistEn2D; 0.03 for Cluster Prominence; to 0.07 for the remaining five GLCM features. Furthermore, Sp ranged from 0.99 for Cluster Prominence to 1.00 for DistEn2D and the remaining five GLCM features. This step allowed for the summarization of the detection accuracy of the radiographic signs of EOTRH syndrome based on the selected entropy measures and GLCM features.

## 4. Discussion

The benefit of using imaging processing in the radiological assessment of EOTRH will come from the enhanced automated detection of early signs of the disease that might be missed by a mere personal evaluation. As the formation of the computed–aided detector is a multi–stage process requiring extensive basic research, the current study presents the second step towards achieving this main goal. In this paper, the novel quantitative description of incisor tooth radiographs was used to enhance the quality of the radiograph to make identifying the radiographic signs of EOTRH syndrome easier, and if possible, to enhance the specific detection of the radiological signs of grade 1, grade 2, and grade 3 EOTRH syndrome.

Recently, filtering algorithms and texture analysis based on the first– and second–order statistics have been successfully used in the digital processing of the equine maxillary incisor teeth radiographs [17]. As the recent report was focused only on maxillary, not maxillary and mandibulary incisor teeth, the current study was focused similarly to provide the appropriate data sets required for the evaluation of similarities. In the previous study, the GLCM application—supported by filtering by the Normalize filter—improved edge delimitation, and was concluded to be the most advisable for the quantification of radiographic signs of EOTRH syndrome [17]. In the current study, the EOTRH grade–related increase level and accuracy of the EOTRH grade differentiation were compared between the previous digital processing approach and the new one, showing a higher slope of the linear regression equations and a higher sensitivity of radiographic sign detection for entropy–based measures than gray–level matrix–based features. As the same radiographs were used in both approaches, one may uphold the current hypothesis, that digital radiograph processing, including filtering and entropy measures extraction, may be considered as the enhancement of the quantitative description of incisor teeth radiographs and the advancement of field dental radiography. The research undertaken meets Zarychta’s [26] statement that the applicability of entropy measures should be evaluated for various specimens and should highlight advances in the development, testing, and application of radiograph–processing algorithms to standard veterinary dental radiography.

In the case of EOTRH syndrome, two opposing pathological processes, resorption and hypercementosis, affect the incisor teeth [5,11]. In both processes, a variable grade of tooth resorption and mild–to–severe hypercementosis were radiographically recognized in most publications devoted to the radiological diagnosis of EOTRH syndrome [5,6,15,49,50]—resorption shows low radiopaque signs, whereas hypercementosis shows high radiopaque signs on the background of the high radiodensity tooth structure [16,17]. Both processes may involve the whole tooth structure, although resorption signs mainly appear in the enamel, cementum, dentine, and pulp cavity [11], whereas hypercementosis is most commonly found in the apex of the tooth, with bulbous enlargements of cement accumulation [51]. The alternating occurrence of radiological signs of resorption and hypercementosis gives a mosaic pattern of the tooth structure that is separable visually [5,6,15] and quantifiable with second–order descriptive statistics [17]. This quantification, based on the Gray–Level Co–occurrence Matrix evaluation, counts the randomness in the radiograph using the differences in the intensity value of the respective pixels or their surroundings [17]. Precisely, the irregularity of pixels in a given window and the likelihood of similarity of these pixels and the pixels of the next window are the basis of the creation of a matrix where the occurrence of a given pixel is counted [52,53]. Contrarily, all currently used entropy measures are calculated directly on the image [24], returning the repeatability of pixel patterns of the image, which in this application, is related to the texture properties of the radiograph [25]. In a currently considered type of application, the values returned from the radiographs are directly related to the predictability or uncertainty of the radiograph’s spatial patterns and are directly related to the radiograph’s irregularity or complexity [29,30,31,32]. Therefore, one may observe that the approaches based on calculating features from the matrix obtained from the processing step applied to the image, such as GLCM, represent the disorder of the intermediate matrix rather than the irregularity of the image [54], whereas, in the case of the EOTRH radiological signs, measures of irregularity and complexity [29,30,31,32] rather than features of disorder [52] of radiograph texture may be extracted as more relevant and insightful. This hypothesis is supported by the current results, which indicate that measures of irregularity and complexity, such as DispEn2D, are more accurate in the differentiation of the EOTRH radiological signs than the features of the disorder, such as Cluster Prominence, Contrast, Difference Average, Difference Entropy, Difference Variance, and Inverse Variance. It should be highlighted that DispEn2D, rather than GLCM features, may be considered in further automated detection developments, which ultimately aim to improve EOTRH detection.

In the current study, the ROIs annotated on the incisor teeth pass the criterion of being a small size image, thus, one may suspect DispEn2D and DistEn2D rather than SampEn2D, FuzzEn2D, and PermEn2D to be effective in this type of application, especially considering that both DispEn2D and DistEn2D have been shown to be the most suitable for the entropy–based texture analysis of small ROIs which were extracted from thermographs in the equine applications of pregnancy [27] and back load [28] detection. Interestingly, in the current study, only DistEn2D, not DispEn2D, demonstrated the most favourable EOTRH grade–related differences. DistEn2D extracted from Normalize filtering output radiographs was the lowest in the EOTRH 0 group, higher in the EOTRH 1 and 2 groups, and the highest in the EOTRH 3 group. One may observe that the concept of counting the amount of similarity between two windows by measuring the distance between the corresponding windows, used in DistEn2D [33,47], is more suitable for the texture analysis of small regions of the radiographs of equine incisor teeth than the concept of using the sigmoid function, as used in DispEn2D [32,45]. The DistEn2D algorithm is invariant to rotation [33,44], whereas the DispEn2D algorithm is the least sensitive to rotation, translation, and image size, out of the five currently studied measures of entropy [45]. Thus, it appears that the concept of a measure, and not the rotation, is essential to the usefulness of the DistEn2D algorithm in this application. These findings justified the choice to use the DispEn2D for further automated detection development.

One may note that the DistEn2D value was lower after Normalize and Median filtering than after Laplacian Sharpening filtering, and the DispEn2D algorithm was the least sensitive to filtering out of the five currently studied measures of entropy [29,30,31,32]. This can be considered a benefit over the GLCM approach. All GLCM features considered in the current study differed between EOTRH grades 0–3 after filtering by the Normalize filter but not after Median and Laplacian Sharpening filtration. The Laplacian Sharpening filter returns the output radiographs with a sharper quality than the input radiograph [55], and has been considered more suitable for the first–order than the second–order statistic extracted from equine radiographs [17]. The Normalize filter increases the contrast of the radiographs [56], which seems to be more favourable for the GLCM approaches than other filtering algorithms [17]. As the GLCM approach returns the spatial distribution of the pixel disorder [57], the output radiograph, after filtering by the contrast–improved algorithm, demonstrates a greater grade of differentiation [58,59]. Contrarily, as the DispEn2D measure returns a quantitative description of the irregularities of the images [33], both the contrast improvement by Normalize filter and the noise reduction by Median [48] may provide a good grade of differentiation for EOTRH radiological signs. One may also observe that for the Normalize filtering output radiograph, the slope value of DistEn2D was higher than the slope value of five from six compared GLCM features. These differences in the slopes of the regression curves indicate a greater increase in the value of the entropy measure than GLCM features with regard to the severity of the radiological signs of EOTRH, and suggest a greater usefulness of DistEn2D than GLCM, which was confirmed by the detection accuracy of EOTRH 0 and EOTRH 3. These findings justified the choice of the Normalize and Median filtering for the further automated detection development.

Finally, one may discern that the proposed method of digital processing of radiographs allows to detect the radiological signs of all grades of EOTRH syndrome, although it does not adequately support the differentiation between EOTRH 0 and 1, EOTRH 1 and 2, as well as EOTRH 2 and 3. Although the DistEn2D–based differentiation between EOTRH 0 and 3 was more accurate than GLCM–based differentiation, further studies on a bigger data set are required to verify the applicability of the proposed algorithms. Within the benefit of using imaging processing in the radiological assessment of EOTRH in horses, the enhancement of the quality of the radiograph to facilitate the identification of identifying the radiographic signs of EOTRH syndrome should be highlighted.

## 5. Conclusions

From the entropy measures recently applied in the equine image analysis, only DistEn2D showed the EOTRH grade–related differences which could be introduced to the advanced veterinary diagnostic procedure for incisor teeth disorders. These observed differences were the least susceptible to the use of radiograph filtering algorithms, returning the same values after Normalize and Median filtering. As the EOTRH grade–related differences were the most favourable for DistEn2D extraction from the Normalize filtering output radiographs, this combination of digital radiograph processing may be carefully advised in equine incisor teeth radiography. Interestingly, the considered GLCM features demonstrated a higher susceptibility for filtering than the entropy measures, showing, similar to DistEn2D, the most favourable differences after Normalize filtering. Moreover, both the evidence of similarity and the highest EOTRH grade–related increase level were noted for DistEn2D and Difference Entropy after Normalize filtering. As these two measures also demonstrated the highest accuracy of the EOTRH grade differentiation, one may suggest that they could be introduced as advancements into the field equine dentistry.

## Figures and Tables

**Table 1:** The comparison of details (linearity of the filter, type of output image, and result of filtering) of three filtering algorithms (Normalize filter, Median filter, and Laplacian Sharpening filter) used in the study.

Filter	Linearity	Output Image	Result
Normalize filter [41]	Linear filter	A rescaled image in which the pixels have zero mean and unit variance	An increase in the contrast of the image
Median filter [41]	Non–linear filter	A recalculated image in which the pixels are represented by the medians of the pixels in the neighbourhood of the input pixel	A reduction in the noise
Laplacian Sharpening filter [42]	Non–linear filter	A produced image in which the pixels are convoluted with a Laplacian operator	A change of the regions of rapid intensity and highlights the edges

**Table 2:** The comparison of details (definition, relations between the values and the irregularity/complexity of the image, and application of measures) of five entropy measures (SampEn2D, FuzzEn2D, PermEn2D, DispEn2D, DistEn2D) used in the study.

Entropy Measures	Definition	Values	Application
SampEn2D [29,44]	The negative natural logarithm of the probability of similarity of patterns of length *m* with patterns of length *m* + 1 $\mathrm{SampEn}2D=-\ln\frac{\Phi^{m+1}}{\Phi^{m}}$	Low: regular patterns or periodic structures, as they have the same number of patterns for both *m* and *m* + 1 High: irregular patterns	A measure of the irregularity in the pixel patterns
FuzzEn2D [30,45]	The negative natural logarithm of the conditional probability $\mathrm{FuzzyEn}2D=-\ln\frac{\Phi^{m+1}\left(r\right)}{\Phi^{m}\left(r\right)}$	Low: regular patterns or periodic structures High: irregular patterns or non-periodic structures	A measure of the irregularity in pixel patterns but using a continuous exponential function to determine the degree of similarity
PermEn2D [31,46]	The concept of counting permutation patterns π , where the permutation patterns are obtained after ordering the positions of the initial image patterns $\mathrm{PermEn}2D=-\frac{1}{\left(n-d_{n}+1\right)\left(m-d_{m}+1\right)}\overset{d_{n}!\times d_{m}!}{\underset{\pi =1}{\sum }}p\left(\pi \right)\ln p\left(\pi \right)$	Low: regular patterns with the pixels always appearing in the same order High: irregular patterns with the highly disordered image pixels	An identification of irregular structure of the image
DispEn2D [32,45]	The conception of using the sigmoid function relies on mapped to c classes and the values of image pixels form $z_{i,j}^{c}=round\left(c\times v\left(i,j\right)+0.5\right)$ , where v(i,j) $$\mathrm{DispEn}2D=-\frac{1}{\left(n-d_{n}+1\right)\left(m-d_{m}+1\right)}\overset{d_{n}!\times d_{m}!}{\underset{\pi =1}{\sum }}p\left(\pi_{v}\right)\ln p\left(\pi_{v}\right)$$	Low: regular patterns with the low probability of dispersion patterns High: irregular image with the high probability of dispersion patterns	An assessment of the regularity of images with no indeterminacy of small–sized images
DistEn2D [33,47]	The amount of similarity between two windows by measuring the distance between the corresponding windows based on the distance matrix used to estimate the empirical probability density function (ePDF) $\mathrm{DistEn}2D=-\sum_{t=1}^{M}p_{t}log_{2}\left(p_{t}\right)$	Low: regular patterns of the small size images High: irregular patterns of the small size images	A quantitative description of the irregularities of the images, taking into account the small size of the image

SampEn2D—two–dimensional sample entropy; FuzzEn2D—two–dimensional fuzzy entropy; PermEn2D—two–dimensional permutation entropy; DispEn2D—two–dimensional dispersion entropy; DistEn2D—two–dimensional distribution entropy.

**Table 3:** The accuracy (Se—sensitivity; Sp—specificity; PPV—positive predictive value; NPV—negative predictive value) of the detection of EOTRH 0 and EOTRH 3 based on the selected entropy measure (DistEn2D—two–dimensional distribution entropy) and the selected Gray–Level Co–occurrence Matrix (GLCM) features (ClusterProminence; Contrast; DifferenceAverage; DifferenceEntropy; DifferenceVariance; Inverse Variance) extracted from the output images filtered by Normalize filter. Three thresholds (mean; mean + SD; mean + 2SD) were used.

Measures	DistEn2D	Cluster Prominence	Contrast	Difference Average	Difference Entropy	Difference Variance	Inverse Variance
**Threshold**	**mean**						
Se	0.50	0.25	0.25	0.25	0.27	0.25	0.25
Sp	0.95	0.99	0.99	0.99	0.99	0.99	0.99
PPV	0.67	0.94	0.94	0.94	0.94	0.94	0.94
NPV	0.90	0.70	0.70	0.70	0.70	0.70	0.70
**Threshold**	**mean + SD**						
Se	0.13	0.17	0.17	0.17	0.22	0.17	0.17
Sp	1.00	0.99	0.99	0.99	0.98	0.99	0.99
PPV	1.00	0.91	0.91	0.91	0.93	0.91	0.91
NPV	0.84	0.68	0.68	0.68	0.58	0.68	0.68
**Threshold**	**mean + 2SD**						
Se	0.00	0.03	0.07	0.07	0.07	0.07	0.07
Sp	1.00	0.99	1.00	1.00	1.00	1.00	1.00
PPV	-	0.67	1.00	1.00	1.00	1.00	1.00
NPV	0.82	0.65	0.66	0.66	0.66	0.66	0.66

## Data Availability

The data presented in this study are available on request from the corresponding author.
