# Correction: Górski et al. Application of Two-Dimensional Entropy Measures to Detect the Radiographic Signs of Tooth Resorption and Hypercementosis in an Equine Model. *Biomedicines* 2022, *10*, 2914

**DOI:** 10.3390/biomedicines11071865

**Published:** 2023-06-29

**Authors:** Kamil Górski, Marta Borowska, Elżbieta Stefanik, Izabela Polkowska, Bernard Turek, Andrzej Bereznowski, Małgorzata Domino

**Affiliations:** 1 Department of Large Animal Diseases and Clinic, Institute of Veterinary Medicine, Warsaw University of Life Sciences, 02-787 Warsaw, Poland; 2 Institute of Biomedical Engineering, Faculty of Mechanical Engineering, Białystok University of Technology, 15-351 Bialystok, Poland; 3 Department and Clinic of Animal Surgery, Faculty of Veterinary Medicine, University of Life Sciences, 20-950 Lublin, Poland; 4 Division of Veterinary Epidemiology and Economics, Institute of Veterinary Medicine, Warsaw University of Life Sciences, Nowoursynowska 159c, 02-776 Warsaw, Poland

## Incorrect Reference

In the original article [1], the reference **[43]** was incorrectly written as **Padhye, N.; Rios, D.; Fay, V.; Hanneman, S.K. *Pressure Injury Link to Entropy of Abdominal Temperature*; Cizik School of Nursing, The University of Texas Health Science Center at Houston: Houston, TX, USA, 2022; *Preprint***. It should be **Flood, M.W.; Grimm, B. EntropyHub: An open-source toolkit for entropic time series analysis. *PLoS ONE* 2021, *16*, e0259448**.

With this correction, the order of references has not been changed. The authors state that the scientific conclusions are unaffected. This correction was approved by the Academic Editor. The original publication has also been updated.
